# A Native Mass Spectrometry-Based Assay for Rapid Assessment of the Empty:Full Capsid Ratio in Adeno-Associated Virus Gene Therapy Products

**DOI:** 10.1021/acs.analchem.1c02828

**Published:** 2021-09-14

**Authors:** Lisa Strasser, Tomos E. Morgan, Felipe Guapo, Florian Füssl, Daniel Forsey, Ian Anderson, Jonathan Bones

**Affiliations:** † National Institute for Bioprocessing Research and Training (NIBRT), Foster Avenue, Blackrock, Dublin A94 X099, Ireland; ‡ Pharmaron, 12 Estuary Banks, Speke, Liverpool L24 8RB, United Kingdom; § School of Chemical and Bioprocess Engineering, University College Dublin, Belfield, Dublin D04 V1W8, Ireland

## Abstract

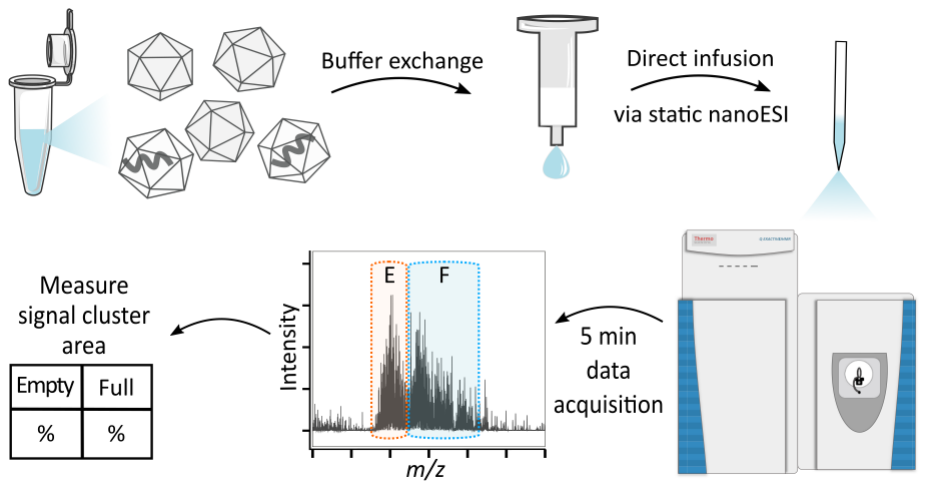

Adeno-associated virus (AAV)-based gene therapy is a rapidly developing field, requiring analytical methods for detailed product characterization. One important quality attribute of AAV products that requires monitoring is the amount of residual empty capsids following downstream processing. Traditionally, empty and full particles are quantified via analytical ultracentrifugation as well as anion exchange chromatography using ultraviolet or fluorescence detection. Here, we present a native mass spectrometry-based approach to assess the ratio of empty to full AAV-capsids without the need for excessive sample preparation. We report the rapid determination of the relative amount of empty capsids in AAV5 and AAV8 samples. The results correlate well with more conventional analysis strategies, demonstrating the potential of native mass spectrometry for the characterization of viral particles.

Adeno-associated virus (AAV)-based gene therapy is evolving rapidly. Since the first AAV-based product was approved by the European Medicine Agency (EMA) in 2012, more than 150 AAV-related clinical trials have been listed on clinicaltrials.gov.^1^ However, despite this impressive progress, analytical methods to monitor quality attributes of recombinant AAV (rAAV)-based products have not advanced with the same speed.

AAVs are composed of a protein capsid that encapsulates a ∼4.7 kb single-stranded DNA genome. The capsid is assembled by 60 copies of the viral proteins VP1, VP2, and VP3 in a ratio of approximately 1:1:10, building a capsid of ∼3.8 MDa.^2^ Of particular concern during the production of rAAV is the amount of empty capsids present, which is not only important for administering the correct dosage but also to account for concerns regarding potential unwanted immune responses caused by empty capsids.^3^ There are various methods available to quantify the amount of empty and full capsids,^4,5^ the most common being analytical ultracentrifugation (AUC)^6^ as well as anion-exchange chromatography (AEX).^7,8^ While these tools have been shown to successfully separate empty and full capsids of various serotypes, absorbance-based methods still face certain limitations. Even though UV detection at 260 and 280 nm can be used to differentiate between empty and full capsids, respectively, during AEX separation, it is known to lack the required sensitivity that is of key importance when working with AAV samples of low concentration. Furthermore, a response factor is needed for correction during quantitation using UV absorbance. This can be avoided using fluorescence detection which, however, does not allow for an unambiguous identification of empty and full capsids.^6,9,10^ This problem could potentially be circumvented using a mass spectrometry (MS)-based approach.

In recent years, the application of mass spectrometry for the analysis of AAV particles has gained an increasing interest.^11,12^ Intact native MS analysis allowed for the determination of the molecular mass of AAV capsids and also revealed the enormous inherent heterogeneity of viral particles.^13^ This heterogeneity in combination with the high molecular weight of intact AAV capsids poses significant analytical challenges. Conventional non-isotopically resolving MS requires the detection and resolution of multiple consecutive charge states for deconvolution and, is therefore, only applicable to samples of limited complexity. While the use of native conditions results in less charges and a higher spatial resolution in the *m*/*z* dimension,^14^ it is currently still not possible to gain charge state resolution for intact AAV capsids.

Even though it is not yet fully commercially available, charge detection mass spectrometry (CDMS) addresses this problem by directly measuring the mass of individual ions, enabling the analysis of high molecular weight species at a level that has not previously been possible.^15−18^ Interestingly, CDMS analysis has shown that empty and full AAV capsid particles have a similar charge state distribution, yet differ significantly in their mass.^16,19^

Here, we exploit this information to determine the empty:full ratio of rAAVs using conventional MS under native conditions. Observed signal clusters derived from empty and full AAV capsids were assigned and facilitated area-based quantification, resulting in an easy-to-implement assay that utilizes standard instrumentation readily available in many characterization laboratories.

## Results and Discussion

**Figure 1:**
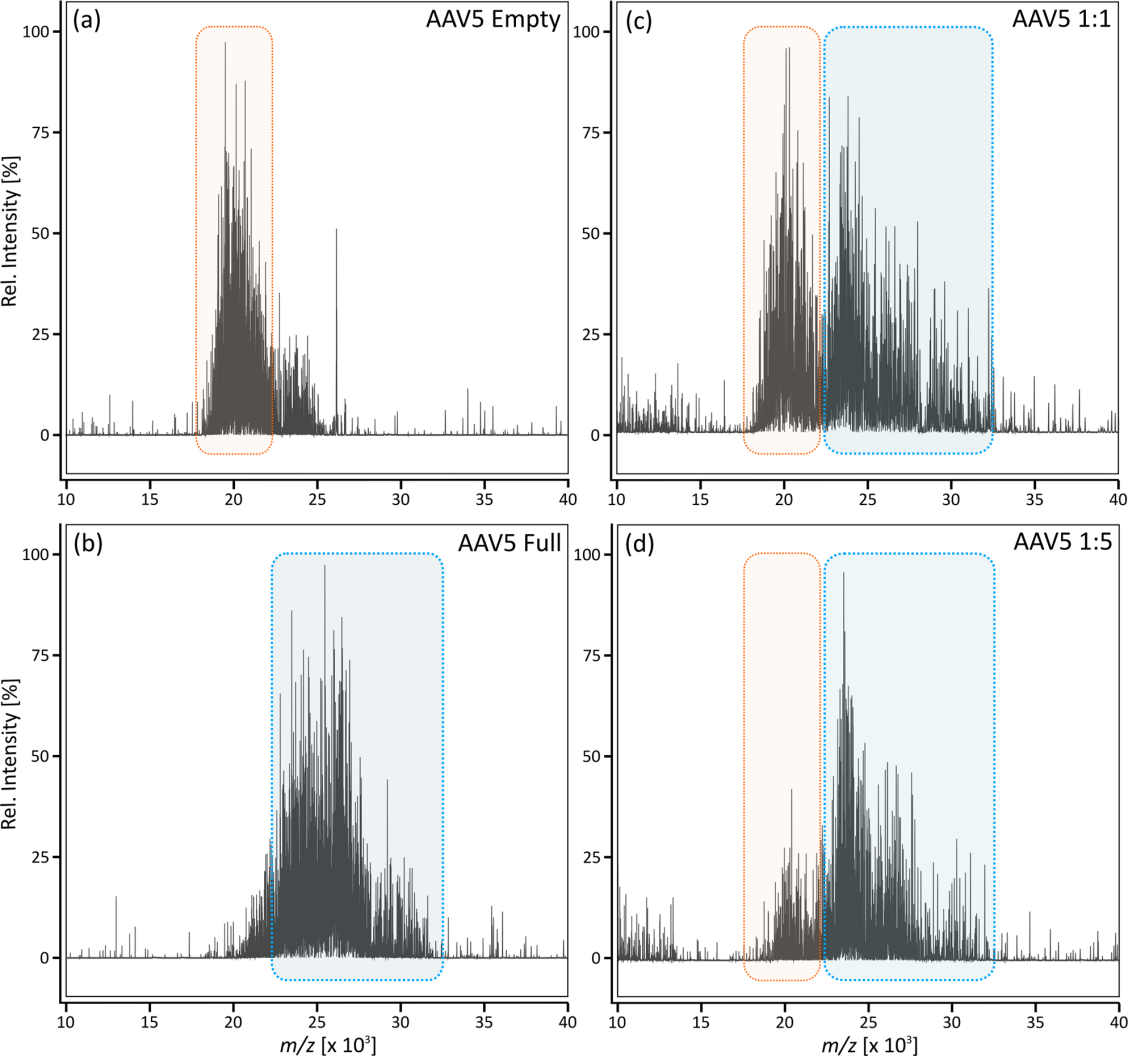
Native direct infusion MS of AAV5. (a) Empty and (b) full AAV5 reference materials were analyzed individually as well as in volumetric mixtures of (c) 1:1 and (d) 1:5. Averaged spectra after 5 min of data acquisition are shown. The signal cluster derived from the empty AAV is highlighted in orange, and that derived from the full AAV is highlighted in blue.

Empty and full AAV5 were analyzed by native direct infusion mass spectrometry, resulting in signal clusters in the range of *m*/*z* 18 000–23 000 and 23 000–32 500, respectively (Figure 1a and b). Importantly, empty reference material might contain full capsids and vice versa, resulting in an additional signal cluster as can be seen in Figure 1a (*m*/*z* > 23 000). Despite this, full and empty capsids appeared to follow the trend previously obtained by CDMS and appeared in different *m*/*z* regions, indicating the same charge while being of different mass.^16,19^ Assuming an average charge state distribution from +150 to +160, as reported previously,^19^ the mass of empty AAV5 was found to be between 2.9 and 3.1 MDa, while full capsids appeared to have a mass of 3.8–4.1 MDa. Thus, the observed mass difference between full and empty particles correlates well with the mass of the incorporated cargo genome (2.5 kb, approximately 800 kDa). Notably, full capsids appeared in a broader cluster, indicating higher heterogeneity due to the incorporated ssDNA.

Next, mixtures of full and empty AAV5 were analyzed. As shown in Figure 1c and d, corresponding to the respective 1:1 and 1:5 mixtures (V/V), observed signal clusters still appeared in the same *m*/*z* region, while the relative abundance changed.

Interestingly, the spray stability during static nanoESI infusion was observed to differ considerably depending on the AAV serotype, with AAV5 being particularly difficult to analyze over extended periods of time. Whether this is due to the sample stability in ammonium acetate requires further investigations. Nevertheless, while the spray stability is a crucial factor during CDMS analysis where extensive data acquisition times are required, it did not noticeably affect the quality of the presented data, as acquisition times were merely 5 min per measurement. To the best of our knowledge, this is the first time spectra obtained from both empty and full AAV5 have been reported.

**Figure 2:**
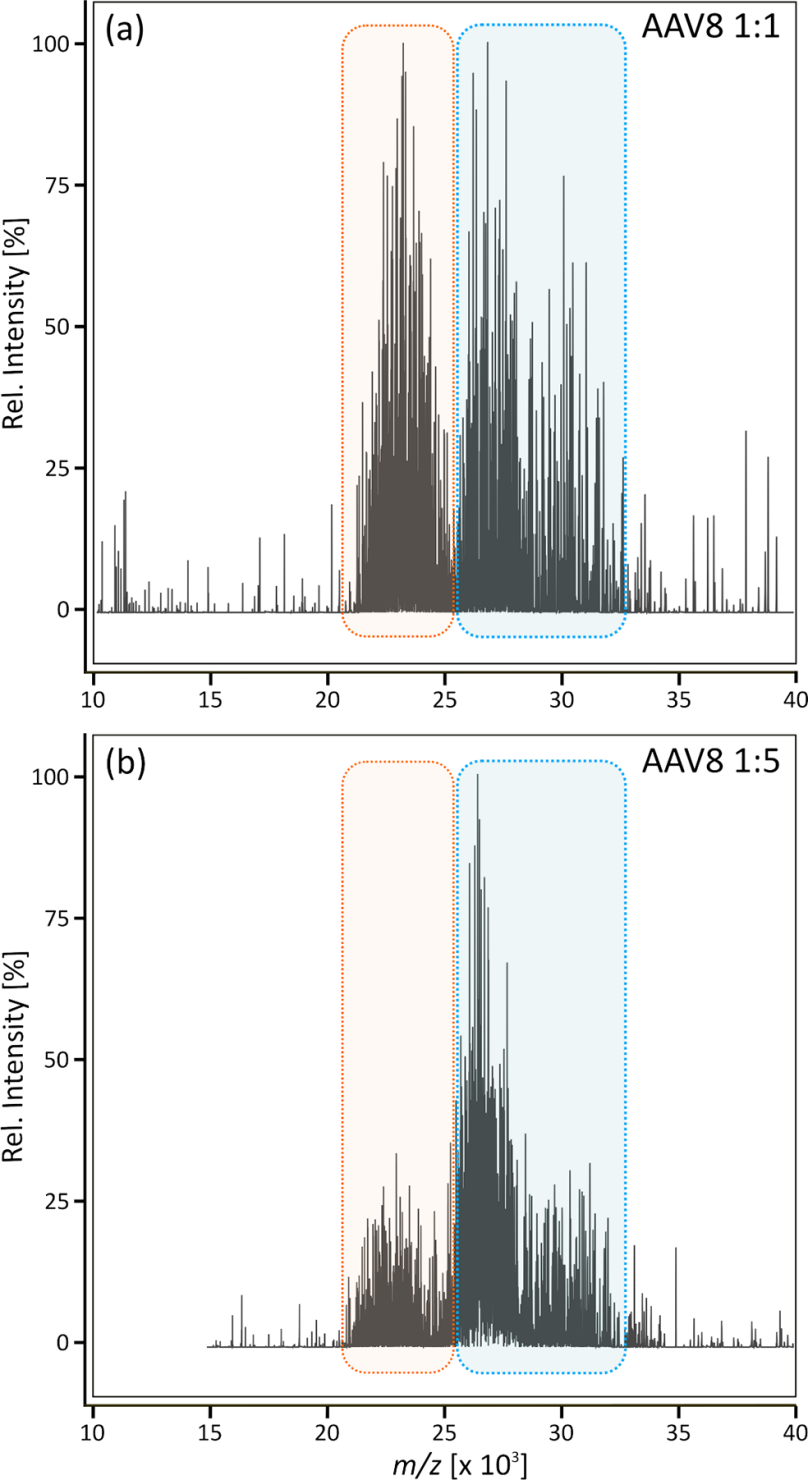
Intact native MS analysis of AAV8. Full and empty reference materials were mixed in ratios of (a) 1:1 and (b) 1:5. The signal cluster derived from the empty AAV is highlighted in orange, and that derived from the full AAV is highlighted in blue.

To test the method for its applicability for different serotypes, the same analysis was performed for AAV8. Figure 2 shows the results obtained for 1:1 and 1:5 mixtures of empty and full capsids of AAV8. The acquired charge upon ionization caused shifts in the *m*/*z* distribution that are dependent on the serotype. Therefore, AAV8 generally appeared at a higher *m*/*z* range, but the intensities corresponding to full and empty particles still changed according to their concentration.

Importantly, the analyses performed did not result in charge state resolution and therefore do not allow for a direct determination of the accurate masses of AAV5 and AAV8. However, differential signal clusters were clear and allowed for the relative quantification of the amount of full and empty capsids. To determine the empty to full ratio of the analyzed samples, corresponding cluster areas were measured using ImageJ. Resulting data were exported for further analysis, and the results obtained are shown in Table 1.

**Table 1:** Empty to Full Ratio Assessment of AAV5 and AAV8^a^

sample	empty:full (V:V)	ratio	% full (MS)	% full (AEX)
AAV5	1:1	1.56	61.02%	62.66%
AAV5	1:5	4.60	82.16%	77.54%
AAV8	1:1	1.41	58.50%	58.21%
AAV8	1:5	3.28	76.62%	73.94%

a The AAV reference material was mixed in a ratio of either 1:1 or 1:5. Samples were analyzed via native MS, and the resulting data were analyzed using ImageJ. Signal clusters corresponding to empty and full capsids were measured and subsequently used to calculate the ratio of empty to full as well as the percentage of full capsids. Additionally, empty and full capsids were separated using AEX, and peak areas were used to calculate the relative amount of full AAV in percent.

As indicated in Table 1, a volumetric mixture of the empty and full reference material in a ratio of 1:1 was found to contain approximately 60% full capsids, while a 1:5 mixture contained about 82% full capsids, which correlates well with what was expected. Furthermore, the obtained results agree with numbers obtained by fluorescence detection using AEX separation (below 5% variation, data shown in Supplementary Figure 1). The reproducibility of the native MS analysis was evaluated by triplicate analysis of AAV5 samples (Figure S2 and Table S1 in the Supporting Information). The standard deviation was found to be below 1.05%, indicating a high consistency. This clearly demonstrates that conventional MS under native conditions can be used to reliably assess the empty to full ratio of AAV samples. Further modifications of the presented method, such as the use of charge reduction to increase the spatial resolution, might also enable the quantification of the amount of partially filled capsids. In any case, the required analysis time is significantly lower compared to that of standard AEX, and the MS analysis furthermore allowed for an unambiguous assignment of signals to full and empty capsids without the need for further experiments in addition to a reliable evaluation of their relative abundances.

**Figure 3:**
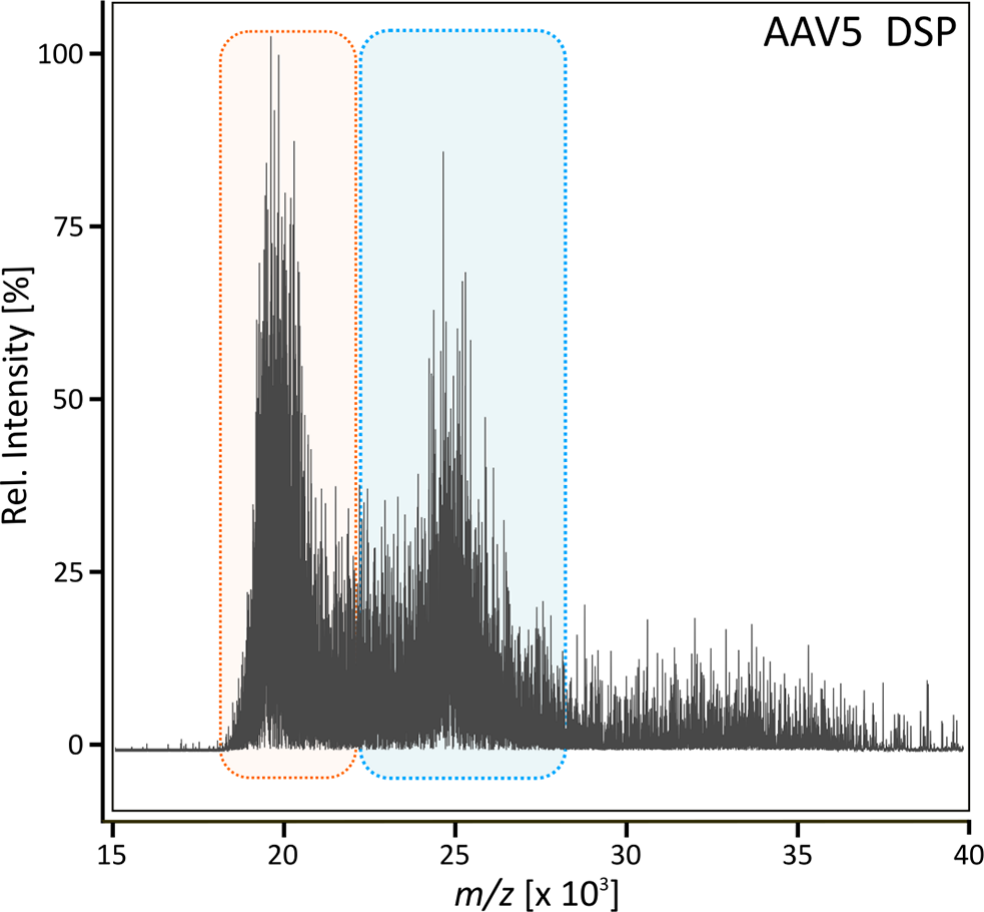
Intact native MS analysis of AAV5 derived from downstream processing (DSP). The signal cluster derived from empty AAV5 is highlighted in orange, and that derived from full AAV is highlighted in blue.

Finally, to demonstrate the applicability of the presented method for samples derived from downstream processing, an in-process sample of AAV5 was analyzed (Figure 3). 53.45% of capsids were found to be full, resulting in an empty:full ratio of 1.15. Results correlate with the amount of full capsids determined via AUC, which was carried out by Pharmaron (data not shown).

## Conclusion

Direct infusion native mass spectrometry was used to measure the relative abundance of AAV5 and AAV8 capsids with and without cargo DNA. Signal clusters derived from empty and full capsids were clearly differentiated, and their relative abundances correlated well with expected values based on the deliberate generation of samples with varying empty:full ratios. Importantly, the approach presented has shown applicability for multiple AAV serotypes, as well as samples derived from downstream processing. Taken together, the results presented clearly demonstrate the potential of using commercially available mass spectrometry for the analysis of critical quality attributes of high-molecular-weight analytes, such as AAVs. Although AAV charge states remained unresolved due to the sample size and complexity, *m*/*z* spacing of the filled and unfilled capsids allowed for relative quantification. Short acquisition times offer great prospect for the coupling of MS analysis strategies to chromatographic separation techniques and the potential for deeper and more accurate analyses will be enabled. Moreoever, further establishment of charge detection MS (CDMS) methods will allow a more accurate mass determination of viral capsids. Further technological developments will fully enable the detailed characterization of next-generation biotherapeutics such as AAV.

## Supporting Information Available

Additional experimental details, materials, and methods about the MS analysis and AEX, results of the empty/full separation using AEX, and replicate analysis of AAV5 using native MS analysis (PDF)

## References

[ref1] Wang D.; Tai P. W. L.; Gao G. Adeno-associated virus vector as a platform for gene therapy delivery. Nat. Rev. Drug Discovery 2019, 18, 358–378. 10.1038/s41573-019-0012-9. 30710128 PMC6927556

[ref2] Vandenberghe L. H.; Wilson J. M.; Gao G. Tailoring the AAV vector capsid for gene therapy. Gene Ther. 2009, 16, 311–319. 10.1038/gt.2008.170. 19052631

[ref3] Gao K.; Li M.; Zhong L.; Su Q.; Li J.; Li S.; He R.; Zhang Y.; Hendricks G.; Wang J.; Gao G. Empty Virions In AAV8 Vector Preparations Reduce Transduction Efficiency And May Cause Total Viral Particle Dose-Limiting Side-Effects. Mol. Ther.--Methods Clin. Dev. 2014, 1, 9 10.1038/mtm.2013.9. PMC4255953 25485285

[ref4] Fussl F.; Trappe A.; Cook K.; Scheffler K.; Fitzgerald O.; Bones J. Comprehensive characterisation of the heterogeneity of adalimumab via charge variant analysis hyphenated on-line to native high resolution Orbitrap mass spectrometry. MAbs 2019, 11, 116–128. 10.1080/19420862.2018.1531664. 30296204 PMC6343805

[ref5] Li T.; Gao T.; Chen H.; Pekker P.; Menyhart A.; Guttman A. Rapid Determination of Full and Empty Adeno-Associated Virus Capsid Ratio by Capillary Isoelectric Focusing. Curr. Mol. Med. 2021, 20, 814–820. 10.2174/1566524020666200915105456. 32933458

[ref6] Burnham B.; Nass S.; Kong E.; Mattingly M.; Woodcock D.; Song A.; Wadsworth S.; Cheng S. H.; Scaria A.; O’Riordan C. R. Analytical Ultracentrifugation as an Approach to Characterize Recombinant Adeno-Associated Viral Vectors. Hum. Gene Ther: Methods. 2015, 26, 228–242. 10.1089/hgtb.2015.048. 26414997

[ref7] Khatwani S. L.; Pavlova A.; Pirot Z. Anion-exchange HPLC assay for separation and quantification of empty and full capsids in multiple adeno-associated virus serotypes. Mol. Ther.--Methods Clin. Dev. 2021, 21, 548–558. 10.1016/j.omtm.2021.04.003. 33997103 PMC8099603

[ref8] Joshi P. R. H.; Bernier A.; Chahal P. S.; Kamen A. Development and Validation of an Anion Exchange High-Performance Liquid Chromatography Method for Analysis of Empty Capsids and Capsids Encapsidating Genetic Material in a Purified Preparation of Recombinant Adeno-Associated Virus Serotype 5. Hum. Gene Ther. 2021, 10.1089/hum.2020.317. PMC10112873 33860673

[ref9] Sommer J. M.; Smith P. H.; Parthasarathy S.; Isaacs J.; Vijay S.; Kieran J.; Powell S. K.; McClelland A.; Wright J. F. Quantification of adeno-associated virus particles and empty capsids by optical density measurement. Mol. Ther. 2003, 7, 122–128. 10.1016/S1525-0016(02)00019-9. 12573625

[ref10] Wang C.; Mulagapati S. H. R.; Chen Z.; Du J.; Zhao X.; Xi G.; Chen L.; Linke T.; Gao C.; Schmelzer A. E.; Liu D. Developing an Anion Exchange Chromatography Assay for Determining Empty and Full Capsid Contents in AAV6.2. Mol. Ther.--Methods Clin. Dev. 2019, 15, 257–263. 10.1016/j.omtm.2019.09.006. 31720304 PMC6838793

[ref11] Dulfer J.; Kadek A.; Kopicki J. D.; Krichel B.; Uetrecht C. Structural mass spectrometry goes viral. Adv. Virus Res. 2019, 105, 189–238. 10.1016/bs.aivir.2019.07.003. 31522705

[ref12] Worner T. P.; Shamorkina T. M.; Snijder J.; Heck A. J. R. Mass Spectrometry-Based Structural Virology. Anal. Chem. 2021, 93, 620–640. 10.1021/acs.analchem.0c04339. 33275424 PMC7807421

[ref13] Worner T. P.; Bennett A.; Habka S.; Snijder J.; Friese O.; Powers T.; Agbandje-McKenna M.; Heck A. J. R. Adeno-associated virus capsid assembly is divergent and stochastic. Nat. Commun. 2021, 12, 1642 10.1038/s41467-021-21935-5. 33712599 PMC7955066

[ref14] Wohlschlager T.; Scheffler K.; Forstenlehner I. C.; Skala W.; Senn S.; Damoc E.; Holzmann J.; Huber C. G. Native mass spectrometry combined with enzymatic dissection unravels glycoform heterogeneity of biopharmaceuticals. Nat. Commun. 2018, 9, 1713 10.1038/s41467-018-04061-7. 29712889 PMC5928108

[ref15] Todd A. R.; Barnes L. F.; Young K.; Zlotnick A.; Jarrold M. F. Higher Resolution Charge Detection Mass Spectrometry. Anal. Chem. 2020, 92, 11357–11364. 10.1021/acs.analchem.0c02133. 32806905 PMC8587657

[ref16] Pierson E. E.; Keifer D. Z.; Asokan A.; Jarrold M. F. Resolving Adeno-Associated Viral Particle Diversity With Charge Detection Mass Spectrometry. Anal. Chem. 2016, 88, 6718–6725. 10.1021/acs.analchem.6b00883. 27310298 PMC6537880

[ref17] Kafader J. O.; Melani R. D.; Durbin K. R.; Ikwuagwu B.; Early B. P.; Fellers R. T.; Beu S. C.; Zabrouskov V.; Makarov A. A.; Maze J. T.; Shinholt D. L.; Yip P. F.; Tullman-Ercek D.; Senko M. W.; Compton P. D.; Kelleher N. L. Multiplexed mass spectrometry of individual ions improves measurement of proteoforms and their complexes. Nat. Methods 2020, 17, 391–394. 10.1038/s41592-020-0764-5. 32123391 PMC7131870

[ref18] Elliott A. G.; Harper C. C.; Lin H. W.; Williams E. R. Mass, mobility and MS(n) measurements of single ions using charge detection mass spectrometry. Analyst 2017, 142, 2760–2769. 10.1039/C7AN00618G. 28636005 PMC12711220

[ref19] Worner T. P.; Snijder J.; Bennett A.; Agbandje-McKenna M.; Makarov A. A.; Heck A. J. R. Resolving heterogeneous macromolecular assemblies by Orbitrap-based single-particle charge detection mass spectrometry. Nat. Methods 2020, 17, 395–398. 10.1038/s41592-020-0770-7. 32152501

