# Supplementary material for: A Native Mass Spectrometry-Based Assay for Rapid Assessment of the Empty:Full Capsid Ratio in Adeno-Associated Virus Gene Therapy Products
Source: Anal Chem. 2021 Sep 14;93(38):12817–21. doi: 10.1021/acs.analchem.1c02828 (PMC8482367; doi:10.1021/acs.analchem.1c02828)
Supplement: Supplementary file 1 — ac1c02828_si_001.pdf [file ac1c02828_si_001.pdf]

Supporting Information

A Native Mass Spectrometry-Based Assay for Rapid Assessment of the Empty:Full Capsid Ratio in Adeno-Associated Virus Gene Therapy Products

Lisa Strasser[1], Tomos E. Morgan[1], Felipe Guapo[1], Florian Füssl[1], Daniel Forsey[2], Ian Anderson[2], Jonathan Bones[1,3*]

[1]NIBRT – National Institute for Bioprocessing Research and Training, Foster Avenue, Blackrock, Dublin, A94 X099, Ireland

[2]Pharmaron, 12 Estuary Banks, Speke, Liverpool L24 8RB, United Kingdom

[3]School of Chemical and Bioprocess Engineering, University College Dublin, Belfield, Dublin, D04 V1W8, Ireland

[*]To whom correspondence should be addressed: jonathan.bones@nibrt.ie, tel: +35312158100, fax: +35312158116

Table of Content:

1. Experimental Section
1.1. Native MS analysis
1.2. Anion exchange chromatography
2. Results:
Supplementary Figure 1 – Anion exchange chromatography-based LC-separation of empty and full AAV capsids.
Supplementary Figure 2 – Replicate analysis of AAV5 using native MS.
Supplementary Table 1 – Empty to full ratio assessment of AAV5.

1. EXPERIMENTAL SECTION

AAV5 and AAV8 full and empty references were purchased from Virovek (Hayward, CA, USA). Both serotypes are derived from Sf9 insect cells using a baculovirus expression system. Ammonium acetate (99.999% trace metals basis), bis-tris propane (BTP) and trimethylammonium chloride (TMA) were purchased from Sigma Aldrich (Wicklow, Ireland) and LC-MS grade Water was obtained from Fisher (Dublin, Ireland).

1.1. Native MS analysis. Prior to analysis, AAV samples were buffer exchanged into aqueous ammonium acetate (100 mM, pH 6.8-7.0) using Bio-Spin® P-6 Gel Columns (Bio-Rad Laboratories, Hercules, CA, USA) according to the manufacturer's instructions. The final sample concentration was between $5 \times 10^{12} – 1 \times 10^{13}$ viral particles/mL, an aliquot of 5 µL was loaded into double-coated borosilicate nESI emitter tips (Thermo Scientific, Hemel Hempstead, UK). Samples were analyzed on a Q Exactive UHMR hybrid quadrupole-Orbitrap mass spectrometer equipped with a Nanospray Flex ion source (Thermo Fisher Scientific, Bremen, Germany). Instrument parameters

were optimized using a sample with known concentration of empty and full capsids to avoid biased results. Data was acquired in positive ion mode using a resolution setting of 25 000 (at *m/z* 200), 10 microscans and an automatic gain control of 1e6 with a maximum injection time of 200 ms. Spray voltage was 1.5 kV, capillary temperature was set to 250°C and the S-lens RF level was 200. The ion transfer target *m/z* and detector optimization were set to "high *m/z*". In-source trapping was enabled with a desolvation voltage of – 100 V and a source DC offset of – 50 V. Extended trapping of particles was carried out in the HCD cell with a HCD energy of 150 V. Sulphur hexafluoride (SF_6) was used as collision gas at 4.0 e-10 mbar (UHV readout). Data was acquired for 5 min with transient averaging enabled.

1.2. Anion exchange chromatography. Empty and full separation of AAV samples was conducted on a ProPac SAX-10 1 × 50 mm column (Thermo Fisher Scientific, Sunnyvale, CA, USA) using fluorescence detection (Ex280/Em340) on a Vanquish Horizon UHPLC system (Thermo Scientific, Germering, Germany). Buffer A was 20 mM BTP at pH 9.0 and buffer B was 20 mM BTP with 1 M TMA at pH 9.0. At a flow rate of 0.15 mL/min and a temperature of 30°C, empty and full capsids were separated using the following conditions: samples were loaded onto the column at 0.1% B followed by an isocratic hold for 3 min. Separation was done using a linear gradient of 1-50% B in 15 min followed by a wash step at 90% B for 1.5 min and column re-equilibration at 0.1% B for 20 min.

2. RESULTS

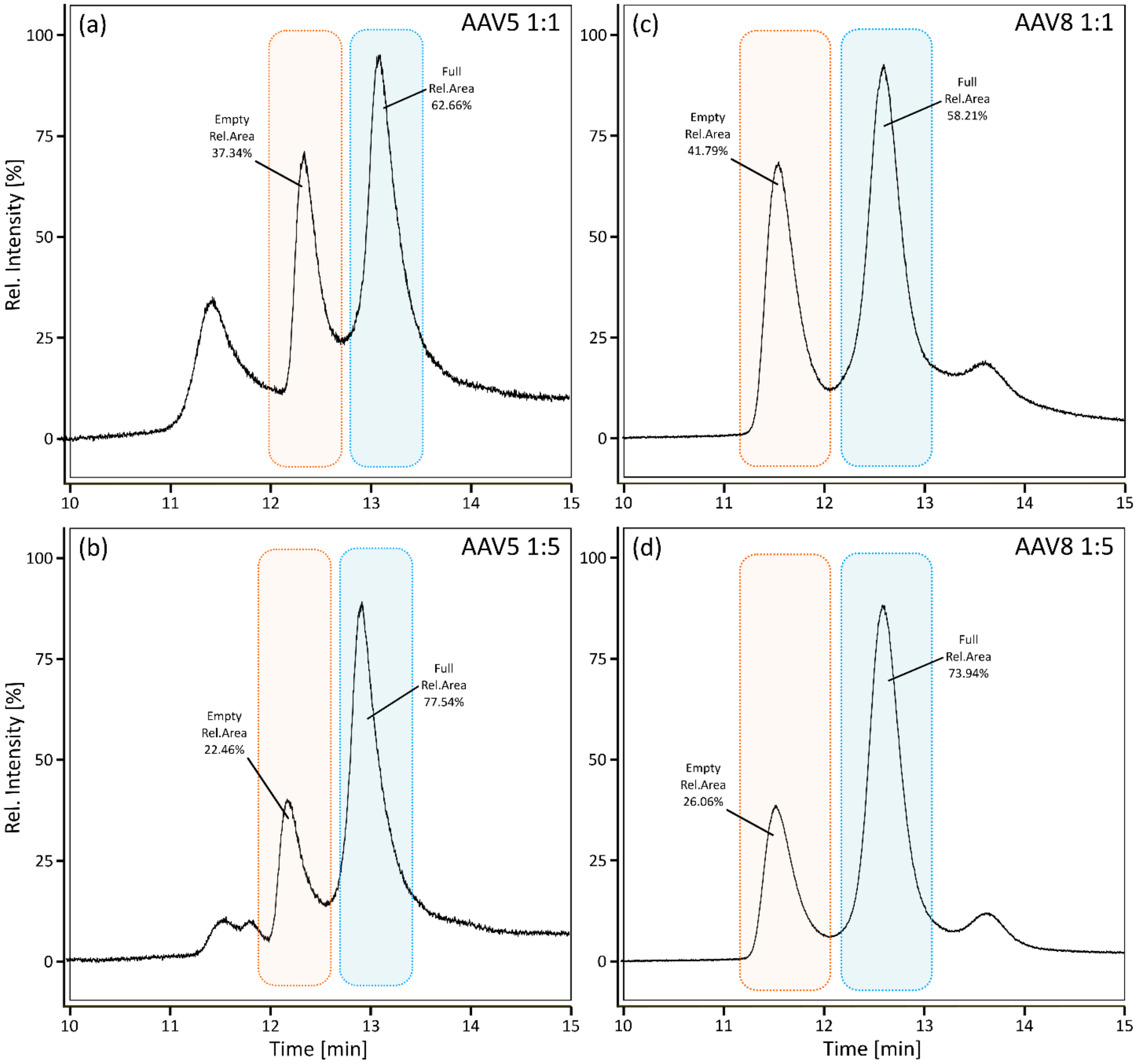

Supplementary Figure 1. *Anion exchange chromatography-based LC-separation of empty and full AAV capsids. AAV5 reference material was analyzed in a volumetric mixture of 1:1 (a) as well as 1:5 (b). Similar analysis was carried out for AAV8 (c, d). Highlighted in orange are peaks derived from empty AAV capsids, blue indicates full capsids. Labels show relative peak area in %.*

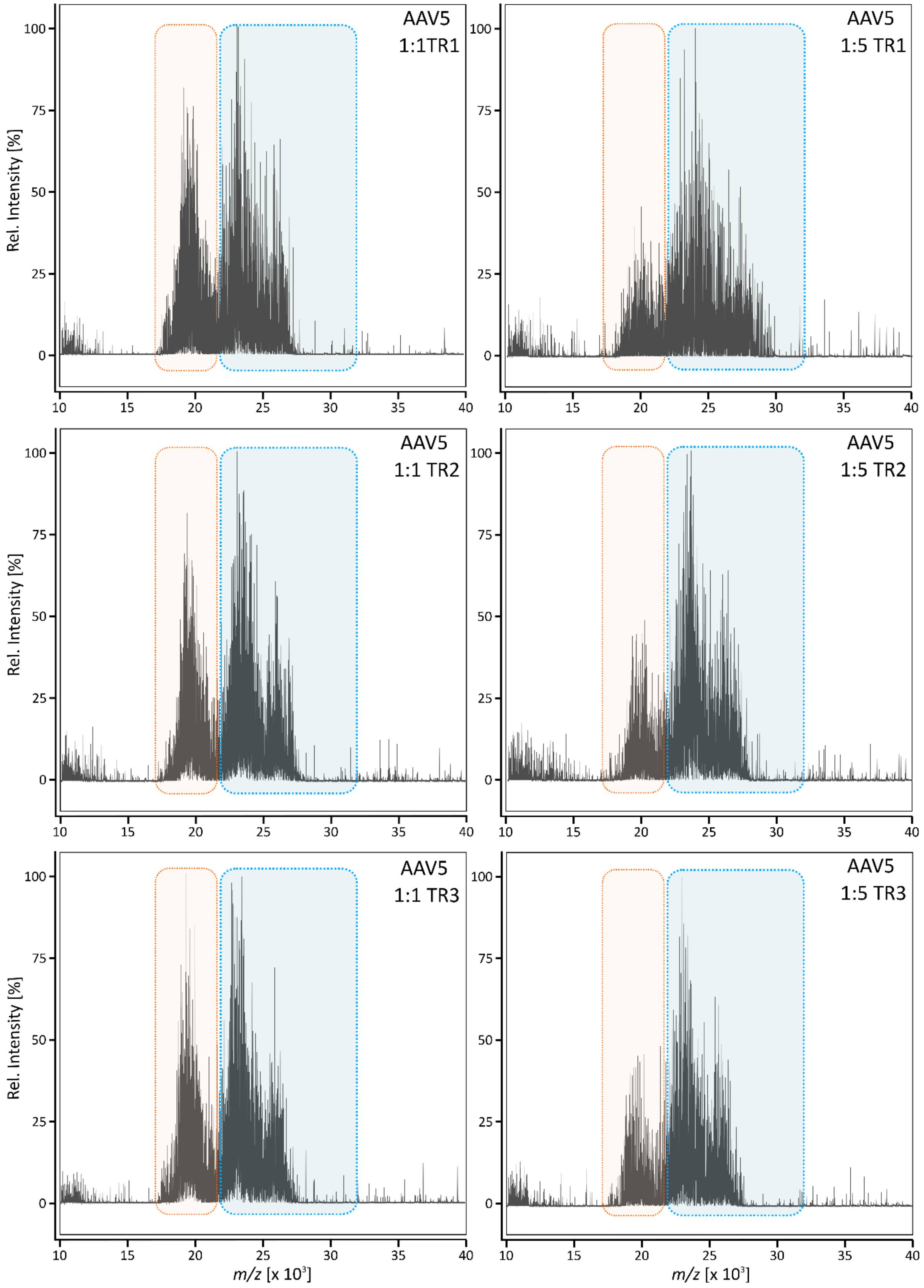

Supplementary Figure 2. *Replicate analysis of full and empty AAV5 capsids using native MS. Signal cluster derived from empty particles are highlighted in orange, full particles are highlighted in blue. Shown are three technical replicates (TR1-3) using a 1:1 or 1:5 mixture of full and empty reference material, respectively.*

Supplementary Table 1. Empty to full ratio assessment of AAV5. AAV reference material was mixed in a ratio of 1:1 and 1:5, respectively. Samples were analyzed in triplicate via native MS and resulting data was analyzed using ImageJ. Signal clusters corresponding to empty and full capsids were measured and subsequently used to calculate the ratio of empty to full as well as the percentage of full capsids. Shown are average values (n=3) and corresponding standard deviation (SD).

**AAV5 1:1**	**Average**	**SD**
% Full (MS)	62.37	0.78
Ratio	1.66	0.06
**AAV5 1:5**		
% Full (MS)	79.26	1.04
Ratio	3.83	0.23
